# Progression of hydroxychloroquine-induced retinopathy after drug cessation

**DOI:** 10.1007/s00417-025-07037-8

**Published:** 2026-01-08

**Authors:** Richard B. Brown, Alice M. Kitay, Matthew Driban, Dinah Zur, Christoph Kniestedt, Daniel Barthelmes, Elham Sadeghi, Jay K. Chhablani, Mayss Al-Sheikh

**Affiliations:** 1 Department of Ophthalmology, University of Pittsburgh School of Medicine, Pittsburgh, PA USA; 2 Department of Ophthalmology, University Hospital Zurich, University of Zurich, Zurich, Switzerland; 3 Talacker Augen Zentrum Zürich (TAZZ), Zurich, Switzerland; 4 Division of Ophthalmology, Faculty of Medical & Health Sciences, Tel Aviv Sourasky Medical Center, Tel Aviv University, Tel Aviv, Israel; 5 Save Sight Institute, The University of Sydney, Sydney, Australia

**Keywords:** Hydroxychloroquine retinopathy, Spectral-domain optical coherence tomography, Retinal toxicity, Lesion progression

## Abstract

**Purpose:**

Hydroxychloroquine (HCQ) is a frequently used drug for a variety of rheumatological conditions. One significant adverse effect is HCQ retinopathy, which irreversibly affects the photoreceptor layer and retinal pigment epithelium, causing paracentral scotomas and potential visual loss. Retinal impairment is irreversible, even after termination of therapy. This study investigates the progression of the retinal destruction after drug cessation.

**Setting/venue:**

The study was performed at the Departments of Ophthalmology at Zurich University Hospital and Tel Aviv Sourasky Medical Center from January 2012 – December 2020.

**Methods:**

In a multicenter retrospective observational design, we analyzed 25 lesions in 18 eyes of 11 patients with HCQ retinopathy. Patients were examined for retinal alterations using spectral-domain (SD) optical coherence tomography (OCT) scans (Heidelberg Engineering, Heidelberg Germany) at first diagnosis of HCQ retinopathy and at least one follow-up (minimum follow up 2 months duration, maximum follow up 5.5 years). Demographics and clinical aspects including age, ethnicity, therapy duration, indication, cumulative dosage, and follow-up interval were recorded. The lengths of the structural damage including ellipsoid zone defect and photoreceptor alterations were analyzed on OCT images.

**Results:**

Demographically, the average patient age was 53.0 years at time of enrollment. All patients were female. The cohort consisted of 54.5% non-Ashkenazi Caucasian and 18.2% Ashkenazi. Roughly half (54.5%) of the cohort held systemic lupus erythematosus (SLE) as a main diagnosis. The average daily dose of HCQ was 310 mg, with an average treatment duration of 15.6 years. Absolute increases in lesion size ranged from 7 to 552 μm (median change 99 μm). The average rate of progression in this cohort was 131.6 μm/year in across all included eyes.

**Conclusion:**

Our data elucidates the progression of HCQ-induced retinopathy in regard to SD-OCT detected structural changes of the retina after drug cessation. The study highlights the pivotal role of an early detection of retinopathy to reduce the potential risk of fovea-threatening progression as an irreversible side effect of the prevalent drug HCQ.

## Abstract (highlights)

**Key messages:**

***What is known*:**

- Commonly used in rheumatological conditions, Hydroxychloroquine (HCQ) can result in an irreversible retinopathy or maculopathy, causing paracentral scotomas and potential visual loss.
- HCQ retinopathy can progress even after drug cessation. Early detection using OCT imaging is crucial to prevent irreversible vision loss.

***What is new*:**

- This study is the first to quantitatively assess lesion progression after HCQ cessation using SD-OCT.
- Progression may occur at a constant or accelerated rate shortly after cessation.
- Baseline lesion size and individual factors may influence the speed of structural damage.

## Introduction

Hydroxychloroquine (HCQ) is widely used as an immunosuppressive treatment of various autoimmune and rheumatological diseases, such as systemic lupus erythematosus (SLE) and rheumatoid arthritis (RA) [1]. The drug modifies several immunological pathways and downstream substrates; however, its exact molecular effect remains unclear [2]. Due to the low cost and rare occurrence of systemic toxicity compared to other immunosuppressants, the drug is frequently prescribed by physicians of many different specialties [2, 3].

**Fig. 1:**
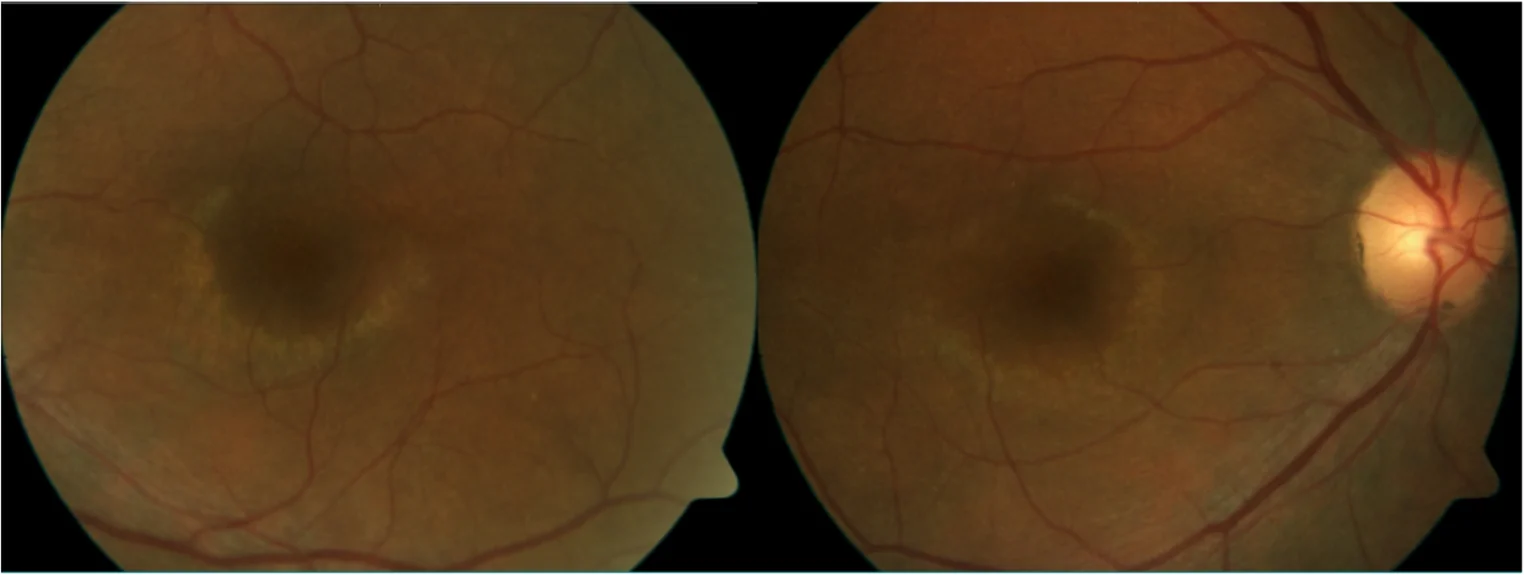
Representative fundus photograph showing retinal changes in HCQ retinopathy. Both photos depict the characteristic ‘Bull’s eye’ pattern of maculopathy, resulting from loss of the photoreceptor inner segment/outer segment junction and pericentral RPE atrophy

HCQ retinopathy is characterized by irreversible changes of the photoreceptor layer and retinal pigment epithelium (RPE), leading to paracentral scotomas and potential blindness [2, 4]. HCQ retinopathy is often asymptomatic with no visual impairment until later stages. The pathognomonic ‘Bull’s eye’ pattern of maculopathy appears due to loss of the photoreceptor inner segment/outer segment junction and pericentral RPE atrophy (Fig. 1), which corresponds to the “flying saucer sign” in OCT scans. This typical finding results from a disruption of the ellipsoid zone in the parafoveal region with an intact outer retina directly under the fovea [5–8]. Recent studies have also shown choroidal degeneration in severe stages [9].

In general, the risk of developing retinal toxicity due to HCQ is low and primarily affects long-term users with a high dosage profile [10]. Risk factors were defined by the American Academy of Ophthalmology (AAO), including duration of intake for more than 5 years, cumulative dose of more than 1000 g, exceedance of 400 mg daily, and concomitant tamoxifen intake [11, 12]. In addition, the existence of retinal degeneration or dystrophy, kidney dysfunction and elderly age have been documented as potential risk factors [11]. Mavrikakis et al. analyzed a prevalence between 0.5 and 2%, whereas studies by Melles et al. reported a risk of more than 7% [13, 14]. Both groups claimed the expected number of unreported cases to be much higher.

Notably, progressive retinal alterations can be seen even after cessation of the drug [8, 15–17]; however, currently, the progression rate after cessation is not known. This retrospective study aims to investigate the progression rate of HCQ retinopathy after drug cessation as well as analyze correlation to risk factors. Understanding the progression of retinal damage after HCQ cessation is critical for optimizing patient management and screening protocols.

Design Multicenter, retrospective cohort study.

## Methods

### Study population

We identified 25 lesions in 18 eyes of 11 patients with HCQ retinopathy from the Departments of Ophthalmology at Zurich University Hospital and Tel Aviv Sourasky Medical Center who visited during the period January 2012 – December 2020 HCQ outpatient clinics. The study adhered to the tenets of the Declaration of Helsinki and patients provided an informed written consent. The study protocol was approved by the Institutional review board of Swiss ethics/BASEC (No. 2019 − 00972).

Inclusion criteria were the following: male or female subjects with an age ≥ 18 years and under treatment with HCQ. Included patients came for regular screening check-ups. Patients were excluded from the data analysis if they possessed other retinal comorbidities, any ocular conditions requiring surgical intervention (e.g., corneal or glaucoma surgery), or an ocular condition which did not allow correct assessment of visual acuity (e.g., after corneal trauma, corneal surgery, damage to anterior segment with severe alterations of the optical system). Patients with insufficient SD-OCT image quality to conduct retinal layer segmentation or those missing follow up OCT data were also excluded from this analysis.

### Data collection

We performed comprehensive ophthalmic examinations for all patients at baseline and follow-up visits, including best-corrected visual acuity assessment, slit-lamp examination, Goldmann tonometry, and fundoscopy. SD-OCT scans (Heidelberg Engineering, Heidelberg Germany) were obtained at first visit where HCQ retinopathy was diagnosed. At least one follow-up OCT after drug cessation was also performed. The minimum follow up duration was 2 months, while the maximum follow up duration was 5.5 years. The demographical and clinical aspects of our patients varied widely in age, ethnicity, therapy duration, indication, cumulative dosage and follow-up interval.

**Fig. 2:**
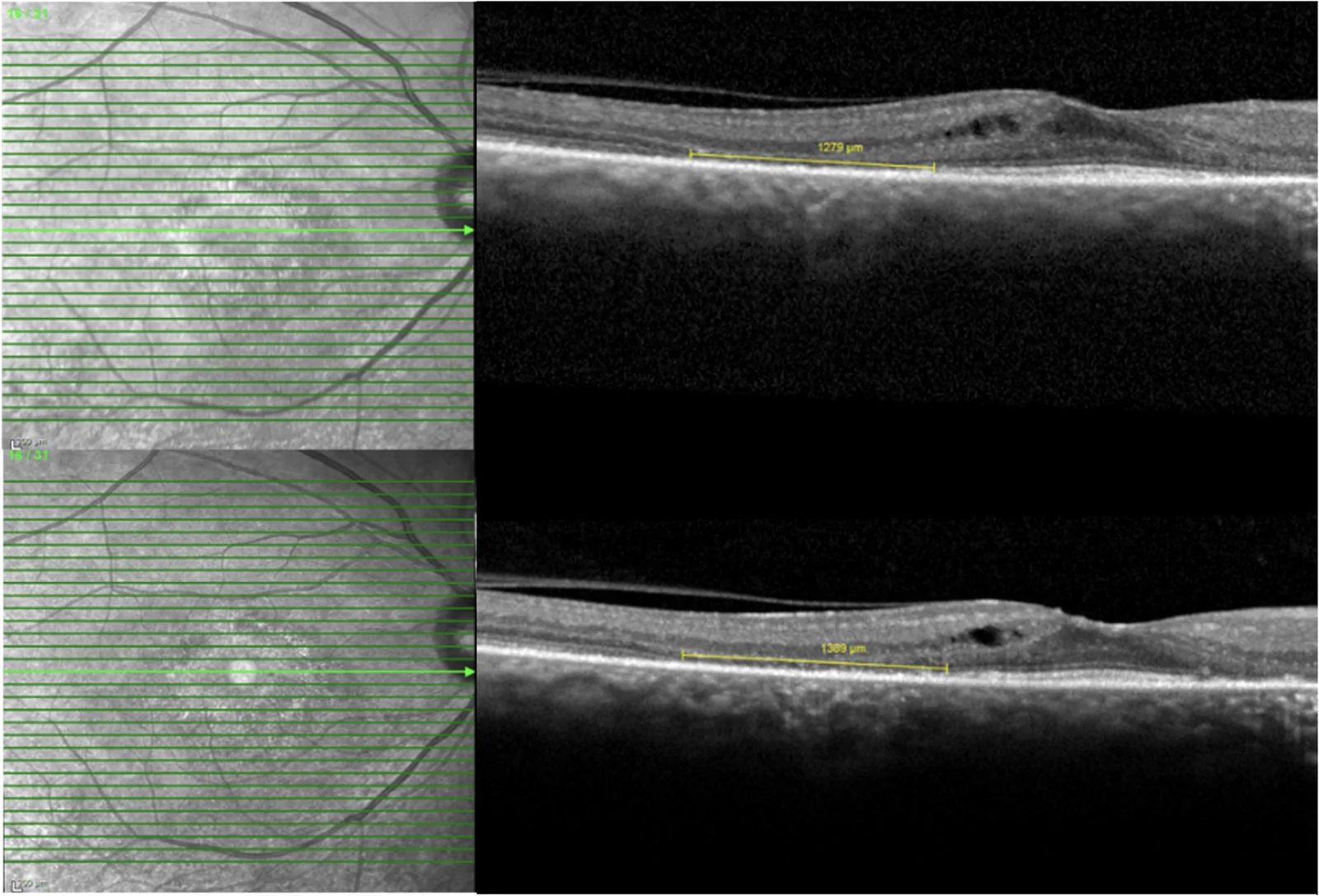
Changes in lesion size between the first examination and follow-up in patients with HCQ retinopathy. The upper panel shows baseline measurements (1279 μm, yellow line) on Optical Coherence Tomography imaging, and the lower panel illustrates the same position during follow-up (1389 μm), highlighting lesion progression. Measurements were conducted using the embedded caliper tool in the HEYEX 2 software (Heidelberg Engineering)

The lengths of the structural disruption or loss of photoreceptor layers damage and ellipsoid zone defect in the parafoveal or pericentral areas on OCT were measured and analyzed. The same scan of the follow-up OCT was then measured at the last clinical exam. To measure the distance of retinal damage, we used the calipers embedded in the image viewer software provided by the Heidelberg software HEYEX 2 (Fig. 2).

To ensure consistency in lesion measurements across different OCT machines and operators, all imaging was performed using Heidelberg Engineering SD-OCT devices. Measurements of the structural damage were conducted using the embedded caliper tool in the HEYEX 2 software, which provides a standardized approach to assessing lesion dimensions. All measurements were independently reviewed by two trained graders, and any discrepancies were resolved through consensus to minimize inter-observer variability.

SD-OCT imaging was chosen due to its superior resolution in detecting structural photoreceptor alterations, particularly in the ellipsoid zone. OCT allows for precise and reproducible linear measurements of lesion extent, which was essential for quantifying progression rates in this longitudinal study. Additionally, SD-OCT was available and performed consistently for all patients during both baseline and follow-up visits. While fundus autofluorescence (FAF) imaging is commonly used to evaluated HCQ retinopathy, this modality was not available for all historical cases.

### Statistical analysis

Statistical analysis was performed using STATA Statistical Software (StataCorp (2019) Release 16. College Station, TX) and Excel (Microsoft (2018), Version 16.16.16). Continuous variables were reported as median and interquartile range (IQR) for skewed data, making these measures more appropriate than means and standard deviations for summarising central tendency and variability. Multivariate regression analyses were performed to determine associations between clinical and demographic variables and lesion progression. A power calculation was not performed due to the retrospective nature of the study and the small sample size. Statistical significance was defined as *p* < 0.05.

## Results

Table 1 illustrates the demographics characteristics of the 11 included patients with HCQ retinopathy.

The average patient age was 53.0 years (range: 29–72 years) on first exam, and 56.0 years (range: 33–74 years) on last follow up. All included patients were female; six patients were diagnosed with SLE,

and two with RA, while the final three patients had Sjögren’s syndrome, scleroderma, and collagenosis, respectively. The cohort consisted of 72.7% Caucasian (54.5% Non-Ashkenazi Caucasian, 18.2% Ashekenazi). Two patients had renal dysfunction, while three had cardiovascular issues. All but three patients reported taking other medications, while no patients were noted to be on concurrent tamoxifen therapy (Table 1). Three patients reported smoking (Patients 9, 10, 11).

**Table 1:** Demographic characteristics of patients with HCQ retinopathy. This table summarizes the demographic and clinical profiles of the 11 included patients, including age, gender, primary diagnosis, and comorbid conditions. Cumulative hydroxychloroquine dose, treatment duration, and daily dose information are also provided. Percentages represent proportions of the total cohort (*n* = 11). Abbreviations: SLE = systemic lupus erythematosus; RA = rheumatoid arthritis; RBW = real body weight

Demographics and Patient Characteristics (*N* = 11)		
Age at First Exam, Average (Range)		53.0 (29–72)
Female, *N*(%)		11 (100%)
Ethnicity, N(%)		
	Non-Ashkenazi Caucasian	**6 (54.5%)**
	Ashkenazi	**2 (18.2%)**
	Other	**3 (27.2%)**
Rheumatologic Diagnosis, N(%)		
	SLE	**6 (54.5%)**
	RA	**2 (18.2%)**
	Other	**3 (27.2%)**
Smoking History, N(%)		**3 (27.2%)**
Cardiovascular History, N(%)		**3 (27.2%)**
Renal Involvement, N(%)		**2 (18.2%)**
Treatment Duration [months], Median (IQR)		**165 (104–205.5.5)**
Daily Dose [mg], Median (IQR)		**325 (200–400)**
Mg/kg, median (IQR)		**4.0 (3.9–5.2)**
Concurrent Use of Other Medication, N(%)		**8 (72.7%)**

Daily dosing was reported in 10 patients (90.9%). Daily dosing ranged from 200 to 400 mg, with 400 mg being the most common dosing scheme (5 patients, 45.5%). Eight patients (72.7%) had weight data available. Dosing ranged from 2.86 to 8.00 mg/kg/day of real body weight (RBW). Two patients were taking HCQ doses above the recommended level of 5 mg/kg/day (5.60 and 8.00 mg/kg/day, Patients 1 and 7, respectively) [11, 18]. Cumulative HCQ dosing ranged from 825 to 3,687 g, with a median of 1,394 g. Median duration of treatment was 171 months (range: 101.6 months – 320 months). Of the 10 patients with available treatment data, all reported taking HCQ for more than five years, and eight patients took the medication for over 10 years (range: 8.5 to 26.5 years). Time between first exam and final follow up ranged from 2 to 82 months (median: 19 months).

### Progression data

**Fig. 3:**
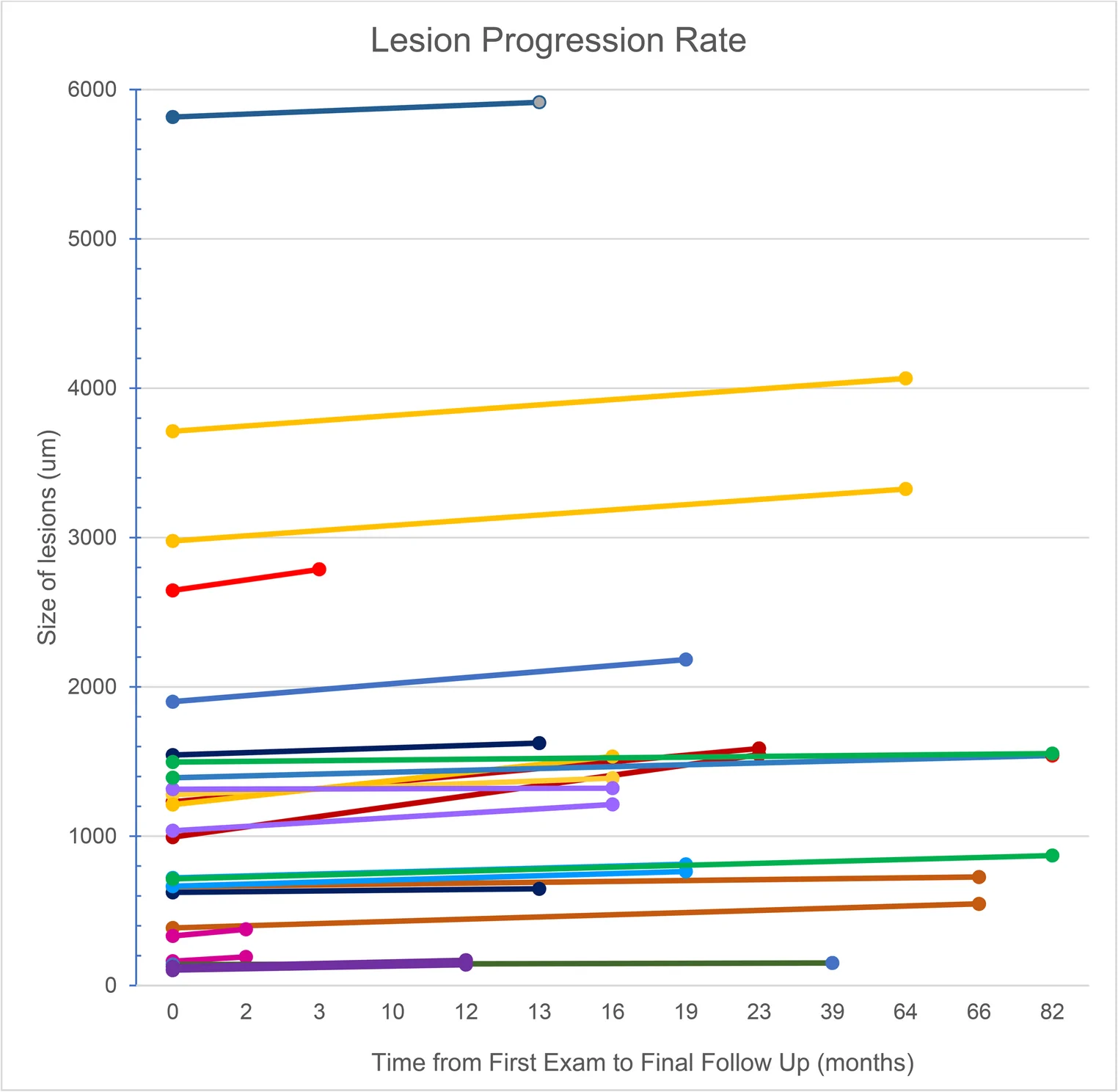
Spaghetti plot illustrating lesion progression rates for each eye of the included patients. Each line represents the lesion progression in one eye of a patient, with colors used to differentiate individual eyes and highlight variability in lesion size progression over time. The y-axis shows lesion size in microns, and the x-axis represents the follow-up period (months)

Twenty-five lesions in 18 eyes were analyzed; the progression of each lesion size is illustrated in Fig. 3. The largest absolute increase in lesion size was 552 μm, while the smallest increase in lesion size was 7 μm. The median change in lesion size on OCT was 99 μm. Within this cohort, the highest rate of progression was 564.0 μm/year. Notably, the time between the first and follow up exams for this patient was 3 months and the absolute change for this patient was 141 μm. Conversely, the lowest rate of change was 3.1 μm/year, with an absolute change of 10 μm. Overall, the average rate of progression in this cohort was 131.6 μm/year in across all included eyes.

Interestingly, the patient with the largest cumulative dose and treatment period (Patient 5, 3687 g over 318 months) exhibited relatively minor lesion progression with all measurements changing less than 100 μm/year (Table 2). This patient also had the largest recorded baseline lesion at 5816 μm.

**Table 2:** Lesion progression rates and baseline lesion characteristics for patients with HCQ retinopathy. The table outlines the progression rates for each individual lesion (absolute change in lesion size in µm divided by time in years between first exam and follow up), as well as the overall average progression rate for each patient (µm/year), their cumulative HCQ dose, and cumulative treatment duration. ID numbers correspond to individual patients, and OD (right eye) and OS (left eye) represent lesion measurements in each eye. Abbreviations: OD = oculus dexter (right eye), OS = oculus sinister (left eye), µm = micrometers, yr = year

Patient	OD 1 (µm/yr)	OD 2 (µm/yr)	OS 1 (µm/yr)	OS 2 (µm/yr)	Patient Avg. Change Rate (µm/yr)	Calculated Cumulative Dose (grams)	Treatment Duration (months)
1	29.3		11.8		20.5	3358.0	270
2			65.3	66.4	65.8	1898.0	150.0
**3**	**168.3**			**288.0**	**237.1**	**1095.0**	**174**
**4**	**174.0**	**270.0**			**222.0**	**-**	**-**
**5**	**22.2**	**73.8**	**91.4**		**62.3**	**3687.0**	**318.0**
**6**	**3.1**				**3.1**	**1506.3**	**198.0**
**7**		**178.1**	**62.5**	**57.5**	**99.2**	**1236.8**	**101.6**
**8**	**82.5**	**241.5**	**132.0**	**5.3**	**11.3**	**1394.0**	**229.0**
**9**		**44.0**		**37.0**	**41.2**	**909.4**	**148.5**
**10**	**564.0**				**564**	**-**	**168**
**11**	**21.8**	**8.2**	**23.0**		**17.7**	**825.0**	**110.0**

In multivariate logistic regression, none of age, treatment duration, daily dose, rheumatologic diagnosis, presence of past medical history, concurrent use of other medications, or size of lesion at baseline were significantly associated with lesion progression. Notably, this lack of statistically significant associations may be attributable to the small sample size; the absence of significance does not necessarily imply absence of association.

## Discussion

Hydroxychloroquine (HCQ) is an effective treatment in a variety of autoimmune conditions and remains a mainstay for the treatment of SLE [19, 20]. Given that the use of this medication is increasing, adequate data on the development and progression of HCQ retinopathy are needed to inform appropriate screening guidelines and prevent the development of irreversible vision loss. OCT imaging techniques represent a valuable tool for the detection of structural changes seen in HCQ retinopathy prior to clinically significant vision loss [21]. This study provides the first report of OCT progression data and analysis of associated demographic factors.

This study comprised of an entirely female cohort, which is in alignment with other published studies regarding HCQ retinopathy [21–23] and the female predisposition to develop autoimmune disorders necessitating this medication [24]. Further, approximately half of included patients in this study had SLE, which is also congruent with other reports in the literature [23]. These findings underscore the importance of monitoring female patients, particularly those with long-term exposure to HCQ.

Previous studies have shown a strong association with adjusted daily HCQ dose [21]. Interestingly, five of these patients developed HCQ retinopathy with dosing regimens that fall below the AAO’s recommended 5 mg/kg/day threshold. In those with dosing regimens above 5 mg/kg/day, treatment duration prior to retinopathy development was more than 10 years, supporting previously reported associations [14]. The patient with the shortest treatment duration (101.6 months) developed retinopathy with the highest real body weight adjusted dose of 8.0 mg/kg/day, but a relatively small cumulative dose of 1236.8 g. Other risk factors previously defined by the AAO are present in this cohort [11]. All patients had a treatment duration of longer than 5 years (range: 101.6–318 months), while seven (63.6%) had cumulative HCQ doses of more than 1000 g, supporting previous reports that HCQ retinal toxicity primarily affects long-term users or those with a high dosage profile [10].

Elderly age has also been documented as a potential risk factor [11); however, in this dataset, large absolute changes in lesion size, lesion progression rates, and patient-averaged progression rates were seen in a variety of ages. The patient with the two largest absolute changes in lesion size (357 and 552 mcm, patient 3) was 74 at the time of enrollment, while the next two highest measurements, 348 and 354 μm, were from a 29-year-old patient. Similarly, high lesion progression rates (> 200 μm/year) were seen in patients ranging in age from 49 to 74, with the highest progression rate, 564 μm/year, seen in a 59-year-old patient. Notably, two of the four patients with change rates over 200 μm/year had relatively short follow-up durations of two and three months, respectively, potentially suggesting that some patients experience an initial rapid period of lesion growth. Interestingly, although patients with renal dysfunction may have a relatively higher risk of developing HCQ retinopathy due to higher blood levels of the drug [14, 25], none of the four with high progression rates had been diagnosed with renal dysfunction. Further, the two patients with renal involvement had relatively small patient-averaged progression rates compared to others in this study at 3.1 and 65.8 μm/year. Additional large-scale studies need to be completed to discover which patient characteristics may contribute to accelerated growth periods in such patients. Although some patients demonstrated relatively rapid progression, no consistent pattern could be identified across clinical parameters, likely due to the small sample size. This highlights the need for larger studies to further elucidate potential risk factors for accelerated progression.

Our findings align with the study by Pham et al., who observed progressive retinal changes in patients even years after HCQ cessation [17]. They reported that progression rates were higher in patients with severe baseline retinopathy, with more advanced cases showing a higher rate of lesion growth. Similarly, our study observed variability in progression rates; While our study also observed variability in progression rates, we found that smaller baseline lesions could exhibit notable early progression, suggesting a potential phase of accelerated structural damage shortly after cessation. This early rapid progression may reflect heightened retinal susceptibility following the removal of HCQ exposure. These findings reinforce the importance of early detection and the need for close monitoring, particularly during the initial post-cessation period.

Previous literature has documented that increased ancillary testing has resulted in an overall increase in HCQ retinopathy detection and reported prevalence [14]. Since the dissemination of the AAO guidelines and re-evaluation of the recommended safe dosing threshold, there has been a sharp decline in patients receiving the 400 mg dose relative to the 200 mg dosing regimen [11, 26]. All patients included here were at or below the AAO recommended maximum daily dose of 400 mg, with 5 patients on lower dosages. As a modifiable risk factor, dosing by weight is of particular interest in relation to retinopathy progression, with several authors debating the utility of real and ideal body weights in these calculations [27]. Notably, decreasing dosing regimens for sight preservation must be balanced with the potential risk of damage to other organ systems [27]. As OCT technology improves and becomes more sensitive in the coming years, we will gain additional data on the structural progression of HCQ retinopathy and elucidate risk factors for higher progression rate.

Some limitations exist in the evaluation of this data. This is a retrospective study with a small sample size, including two eyes of patients; studies with larger sample sizes may provide the statistical power needed to define more concrete correlations regarding HCQR progression among specific patient demographics. It should be noted that the lack of statistically significant associations may be attributable to the small sample size, which limits the statistical power of multivariate analyses. Therefore, absence of significance does not necessarily imply absence of association. As a relatively rare condition, enrolling sufficient numbers of HCQ retinopathy patients at a single center is challenging. Future longitudinal studies, involving multiple academic centers, are needed to confirm our results and note further associations in the data. In addition, SD-OCT imaging techniques were used in this study; technical advances in this technology may provide higher-resolution imaging and afford insights into disease pathogenesis and the generation of clinically relevant biomarkers. A recent study by Yusuf et al. outlined the utility of novel imaging techniques in the earlier detection of HCQ retinopathy. The use of OCT angiography, quantitative autofluorescence, fluorescence lifetime imaging ophthalmoscopy, widefield OCT, and minimum intensity analysis, among others, may decrease the clinical sequelae of HCQ retinopathy by detecting retinal generation prior to the development of visually significant retinopathy [28]. With a proportionally wider area of the retina imaged, we can also gain a deeper understanding of the temporal patterns of regions and retinal layers affected. Accordingly, additional studies documenting retinopathy progression after drug cessation using different imaging modalities may be helpful in determining key risk factors [27].

## Conclusion

In conclusion, this longitudinal SD-OCT imaging data describes the structural progression of HCQ retinopathy after drug termination. Prompt and early detection of retinopathy can reduce the potential for irreversible vision loss secondary to a prevalent anti-sight-threatening risk of fovea-threatening progression as an irreversible side effect of this prevalent immunosuppressive medication. Future studies with larger cohorts and advanced imaging modalities are necessary to further elucidate progression mechanisms and refine patient monitoring strategies.
